# Central processing of afferent renal pathways—significance and implications

**DOI:** 10.1007/s00424-020-02462-6

**Published:** 2020-09-12

**Authors:** Kristina Rodionova, Roland Veelken

**Affiliations:** 1 Department of Internal Medicine 4 - Nephrology and Hypertension, Friedrich-Alexander University Erlangen, 91054 Erlangen, Germany; 2 Department of Internal Medicine 4 - Nephrology and Hypertension, Paracelsus Private Medical School Nuremberg, Nuremberg, Germany

Fen Zheng and his co-authors investigated in their article *Interleukin-1β in hypothalamic paraventricular nucleus mediates excitatory renal reflex* the central pathways of a sympathetic excitatory reflex, which is triggered by renal afferent nerve fibers. The authors had already described this reflex in more detail before: a direct infusion of capsaicin into the renal interstitium resulting in an increase of sympathetic nerve activity to the kidneys. In their recent work addressing central nervous mechanisms, the authors focused on the paraventricular nucleus of the hypothalamus, which plays an important role in the processing of various peripheral afferent information, which in turn participate in the generation of central sympathetic outflow. With regard to their specific question, the authors convincingly demonstrated that sympathetic activation following administration of capsaicin directly into the renal interstitium is mediated via IL-1β in the paraventricular nucleus of the hypothalamus.

The investigation of neurophysiological structures like the paraventricular nucleus of the hypothalamus is important because the central pathways integrating afferent and efferent renal nerve activity are not well understood. Clinical studies suggest an important role of renal sensory afferent innervation in the regulation of efferent sympathetic tone. In patients with hypertension and end-stage kidney disease, peripheral sympathetic activity remained increased after renal transplantation. Peripheral sympathetic tone decreased only in patients who had additionally undergone bilateral nephrectomies of the failing kidneys [7].

At first glance, it may seem surprising why an interleukin-like IL-1β should play such a prominent role in processing afferent neuronal input for the generation of central sympathetic outflow. However, one should bear in mind that the immune system and many visceral organs are autonomously innervated quite densely [8]. The influence of the autonomous innervation on acute and chronic inflammation with respect to various organs like liver, skin, the joints, and kidneys has been repeatedly reported [1, 2, 10]. The matter has become more intriguing since afferent renal nerve fibers will not only influence central sympathetic outflow [4]. Rather, afferent nerve fibers in general are also known to release neuropeptides like CGRP and SP influencing local circulation and interfering with immune processes [3]. Hence, catecholamines as well as the mentioned peptide transmitters may contribute to neuroimmune interactions while also influencing the activity of efferent sympathetic and afferent pathways. In this respect, pro-inflammatory cytokines like IL-1β were described to influence the central nervous system via the circulation or stimulate peripheral afferent nerve fibers [6].

If capsaicin was not injected into the renal tissue but directly into the organ via the renal arteries, a long lasting sympathetic depression [5] and no sympathoexcitation occurred as described by Fen Zheng and co-authors. The different results are probably due not only to different routes of administration but also touch on the still unsolved question of how renal afferences actually influence the sympathetic nervous system: While decreases in peripheral sympathetic activity after nephrectomy favor sympatho-excitatory afferents from the kidneys [7], we are also presented with further reports that rather support a role of afferent nerve fibers in sympatho-depression [4]. One special example known for a long time are reno-renal sympatho-depressory reflexes which are impaired in spontaneously hypertensive rats [9].

More extensive research will be needed to clarify these last mentioned open points. The fact that there will be no quick and easy explanations in this context is not least due to the observation that afferent nerve fibers always have both mechano- and chemosensitive properties, whereby they express a large number of different receptors, quite a few of them with significant involvement in immunological responses.
